# Editorial: Analytical devices based on immobilized macromolecules for structural and activity/affinity studies in drug discovery

**DOI:** 10.3389/fmolb.2022.971076

**Published:** 2022-10-20

**Authors:** Caterina Temporini, Enrica Calleri, Manuela Bartolini, Marcela Cristina de Moraes

**Affiliations:** ^1^ Department of Drug Sciences, University of Pavia, Pavia, Italy; ^2^ Department of Pharmacy and Biotechnology, Alma Mater Studiorum, University of Bologna, Bologna, Italy; ^3^ BioCrom, Departamento de Química Orgânica, Instituto de Química, Universidade Federal Fluminense, Niterói, Brazil

**Keywords:** immobilized enzyme reactors, bioaffinity chromatography, binding assay, drug discovery, structural elucidation

The immobilization of macromolecules on chromatographic supports, namely bioaffinity chromatography, was proposed in 1969 by Soczewinsky and Bieganowska (Soczewiński and Bieganowska, 1969). The idea of usingon-flow analytical systems to model a biological event is based on the fact that the same interactions occur in a biological environment and at the molecular scale (i.e.; hydrogen bonding, electrostatics, van der Waals forces, and *π*-π interactions), which is the basis of chromatographic separation (de Moraes et al., 2019). Immobilized enzymes, proteins, and receptors have been used to study kinetic mechanisms and to characterize ligand-receptor binding interactions at the initial stages of drug discovery as well as during drug development.

In this Research Topic, one review and three original research papers provide examples of the most recent and innovative applications of analytical systems based on immobilized macromolecules to study the structure, activity, and affinity of molecules of pharmaceutical interest. Each published manuscript is presented and briefly described below.

Glycosylation is one of the most relevant post-translation modifications in proteins, affecting both the half-life and biological activity. As such, several studies have focused on the development of analytical methodologies to understand the qualitative and quantitative characteristics of glycosylation in proteins. Many of these studies utilize an initial on- or off-line step to selectively enrich glycosylated proteins or glycopeptides, coupled with several separations and detection methods. One of the most common enrichment methods is the use of immobilized lectins proteins, which have an affinity for the specific sugar moieties found in glycoproteins. The review by Goumenou et al. presents different methodologies for the use of lectins in both affinity chromatography and solid-phase extraction, allowing fast and automated glycosylation analysis. The authors discuss state-of-the-art use of commercial and homemade lectin–based affinity sorbents for the extraction and enrichment of glycoproteins and glycopeptides.

A promising approach for high-throughput screening in the early stages of drug discovery is to utilize on-line activity assays. In an interesting study, (Seidl et al.) describe a unique platform for the on-line screening of acetylcholinesterase (AChE) inhibitors. The authors employed a combination of reverse-phase separation, using a capillary immobilized–enzyme reactor (IMER) in a 2D liquid chromatographic system, coupled with mass spectrometry detection (LC-MS). In this approach, an analytical column was used in the first step to separate the sample, after which, the formed product was detected by MS in the second step. Each fraction from the first step was combined with the enzyme substrate using a syringe pump and subsequently screened for its potential to inhibit the AChE enzyme. A mixture containing three known inhibitors, including tacrine, galanthamine, and donepezil was employed to validate the platform. Finally, an ethanolic extract obtained from dry *Hyppeastrum caplyptratum* bulbs was investigated as a proof of concept for the applicability of the developed platform. The described approach represents a major advance in screening AChE inhibitors in natural products, since it avoids the need for pre-treating samples in a fully automated assay.

Monoclonal antibodies (mAb) are one of the most widespread biopharmaceutical products, clinically used in a large variety of illnesses such as cancer, inflammation, diabetes, cardiovascular and genetic disorders, autoimmune diseases, and other infections. The complex heterogeneous structure of mAbs makes them challenging to produce and characterize. Most current analytical methods for the in-depth structural characterization of mAb entail a preliminary structural simplification using enzymatic treatments. This digestion method can be conducted using a middle-up approach. The initial formation of 25–50 kDa mAb fragments by enzymatic treatment used proteases such as IdeS or papain, followed by further analysis by LC-MS or capillary electrophoresis (CE)-MS. In this way, Rinaldi et al. describe the preparation of IMERs based on papain, which can support the analytical characterization of mAb. Two monolith IMERs were prepared through covalent immobilization of papain in either a commercial or homemade column. Nα-benzoyl-l-arginine ethyl ester (BAEE) was employed as a standard substrate for the characterization of both IMERs using an on-flow system. The two IMERs were utilized to structurally characterize the digestion of rituximab (RTX) by LC-MS, allowing reduced enzyme consumption and improved repeatability, compared with off-line approaches, with minimal operator manipulation.

It has been postulated that the fragile histidine triad (FHIT) protein may function as a tumor suppressor, suggesting that it may play a role for the FHIT protein in the carcinogenesis process. Gaudio et al. demonstrated that FHIT binds to and relocates annexin A4 (ANXA4) from the plasma membrane to the cytosol in paclitaxel-resistant lung cancer cells, restoring their chemosensitivity to the drug (Gaudio et al., 2013). The authors also identified the smallest protein sequence in FHIT (ranging from position 7–13) that interacts with ANXA4. This sequence was not only able to bind to ANXA4 but was also able to hold its target in the cytosol during paclitaxel treatment, avoiding ANXA4 translocation inside the cell membrane (Gaudio et al., 2016). Considering FHIT mimetic peptide 7–13 as a potential candidate for the development of therapeutic chemical compounds targeting ANXA4, Scala et al. describe a systematic structure-activity relationship (SAR) that pinpoints the key residues involved when FHIT binds to ANXA4. Different biophysical techniques such as differential scanning fluorimetry, surface plasmon resonance, and microscale thermophoresis were used to study the binding of seven FHIT-derived peptides, synthetized using an Ala-scan approach, to ANXA4. Conformational studies employing circular dichroism and nuclear magnetic resonance were carried out to characterize the synthesized peptides. Lung cancer cell-based investigations allowed identification of one particular peptide that was able to bind to ANXA4 and reduce cell viability in paclitaxel-treated lung cancer cells.

These manuscripts will inspire readers to apply diverse analytical techniques towards advanced applications in medicinal chemistry. Researchers in universities and pharmaceutical industries can exploit increased efficiencies, reliable screening, and structural assays by exploring the considerable potential of immobilized biomolecules associated with various analytical methodologies.
